# Entropy-Based Volatility Analysis of Financial Log-Returns Using Gaussian Mixture Models

**DOI:** 10.3390/e26110907

**Published:** 2024-10-25

**Authors:** Luca Scrucca

**Affiliations:** Department of Economics, Università degli Studi di Perugia, Via A. Pascoli 20, 06123 Perugia, Italy; luca.scrucca@unipg.it; Tel.: +39-075-5855231

**Keywords:** Gaussian mixture models, financial log-returns, volatility, entropy estimation, risk measures

## Abstract

Volatility in financial markets refers to the variation in asset prices over time. High volatility indicates increased risk, making its evaluation essential for effective risk management. Various methods are used to assess volatility, with the standard deviation of log-returns being a common approach. However, this implicitly assumes that log-returns follow a Gaussian distribution, which is not always valid. In this paper, we explore the use of (differential) entropy to evaluate the volatility of financial log-returns. Estimation of entropy is obtained using a Gaussian mixture model to approximate the underlying density of log-returns. Following this modeling approach, popular risk measures such as Value at Risk and Expected Shortfall can also be computed. By integrating Gaussian mixture modeling and entropy into the analysis of log-returns, we aim to provide a more accurate and robust framework for assessing financial volatility and risk measures.

## 1. Introduction

Financial markets are complex and unstable systems where the understanding and the prediction of price movements is crucial for investors, policymakers, and risk managers. Volatility, which refers to the degree of variation in a financial instrument’s price over time, is a key measure of risk in financial markets. Traditional measures of volatility, such as standard deviation and variance, provide valuable insights but often fail to capture the multifaceted dynamic nature of financial data. Evaluating volatility in financial markets is crucial for risk management, helping investors to assess and mitigate potential losses, but also for option pricing models that rely on volatility to determine option values, and in general to make informed decisions regarding asset allocation and portfolio diversification.

Various methods can be employed to assess the volatility of a financial asset, drawing from some or all of the standard daily data, such as opening, high, low, and closing prices. Vințe et al. [1] present an overview of several volatility indices, including a discussion of their limitations. Among these, a standard method to quantify the volatility is to compute the standard deviation of the log-returns of an asset over a specified period [2].

If $P_{t}$ represents the price of a traded stock for the current time frame (a day, for instance), and $P_{t-1}$ is the price of the previous time frame, then the log-return at time *t* can be calculated as

(1) $$y_{t}=\log\left(\frac{P_{t}}{P_{t-1}}\right).$$

Log-returns are widely used in financial econometrics due to their desirable statistical properties, including the approximation of normality for high-frequency data and the simplification of multiplicative processes.

Historical volatility is defined as either the annualized variance or standard deviation of log-returns:

$$s=\sqrt{\frac{1}{n-1}\sum_{t=1}^{n}{\left(y_{t}-\bar{y}\right)}^{2}},$$

where $\bar{y}$ is the mean of the log-returns over the period.

As mentioned in (Arratia [3], Section 2.6), since real financial data often have a mean close to 0, a non-centered standard deviation such as

$$s_{0}=\sqrt{\frac{1}{n-1}\sum_{t=1}^{n}y_{t}^{2}},$$

which requires one less parameter to estimate, provides an estimator with a smaller Mean-Squared Error (MSE) [4].

The use of the standard deviation of log-returns is a common method for assessing volatility and quantifying the risk of an asset’s returns over a specified period. While this approach has several advantages, it also has some drawbacks. In practice, stock returns often exhibit fat tails (higher probability of extreme events) and skewness, indicating that they are not perfectly normally distributed. This can lead to a potentially misleading assessment of risk [5]. Additionally, the calculation of the sample standard deviation is highly sensitive to the presence of extreme values in the data set. A few outliers can disproportionately impact the calculated volatility, potentially leading to a distorted view of the asset’s typical risk profile. In periods with extreme returns (either high gains or losses), the standard deviation might not accurately reflect the true underlying volatility.

Entropy, a concept rooted in information theory [6], is a powerful tool for quantifying the uncertainty in complex systems. Introduced by Shannon [7], entropy measures the average amount of information produced by a stochastic process. In the context of financial markets, entropy can be used to evaluate the unpredictability of log-returns, providing a complementary perspective to traditional volatility measures. It has been observed that entropy incorporates more detailed information about the underlying data distribution, as it captures aspects related to higher-order moments beyond those considered by standard deviation, providing a more comprehensive picture of volatility [8]. Moreover, entropy-based measures can capture nonlinearities and complex dependencies in financial data, which are often overlooked by conventional volatility metrics. Moreover, entropy is adaptable to various time scales, making it a versatile tool for both short-term and long-term volatility analysis.

Entropy-based measures of uncertainty and volatility have found significant applications in finance, for instance, in portfolio selection and asset pricing [9]. More recently, Sheraz and Nasir [10] applied information-theoretic measures in GARCH-type models to capture stock market volatility more accurately. Drzazga-Szczęśniak et al. [11] proposed an entropy-based theoretical framework to describe the behavior of financial time series, particularly under the influence of extreme events. In another contribution, Wang et al. [12] compared Shannon and Rényi entropies with the capital asset pricing model (CAPM) beta for assessing market risk. Their experimental results demonstrated that entropy-based measures offer superior explanatory power in capturing market risk compared to CAPM beta, suggesting that entropy can be a more effective tool for risk assessment. However, these applications typically rely on calculating the entropy from a preliminary density estimation step, which can be obtained using a discretized distribution, such as a histogram, or through a nonparametric kernel density estimate (KDE). These methods introduce significant limitations, as they require tuning hyperparameters, such as the number of bins in histograms or the kernel bandwidth in KDE. The absence of universally accepted rules for selecting these hyperparameters can lead to variability in results and potential biases.

In this paper, we explore the application of Gaussian mixtures for modeling the empirical distribution of financial log-returns and deriving traditional risk measures. We also present a mixture-based estimation of entropy to assess the volatility of financial assets, highlighting its advantages over conventional volatility measures and its potential applications in financial modeling. In Section 2, we introduce the Gaussian mixture statistical framework used to model financial data. This includes the estimation of unknown parameters, the selection of model complexity, and the regularization method required to prevent degeneracies in the fitting algorithm. Moments of Gaussian mixtures are obtained, enabling the computation of risk measures such as Value at Risk (VaR) and Expected Shortfall (ES). Furthermore, we propose an entropy-based approach for assessing the volatility of financial instruments. Section 3 presents some empirical analyses that illustrate the advantages of the proposed approach. Section 4 concludes the paper with a discussion of key findings and potential directions for future research.

## 2. Materials and Methods

### 2.1. Modeling Log-Returns Using Gaussian Mixtures

Gaussian mixture models (GMMs) provide a flexible semiparametric methodology for density estimation. In this approach, a probability density function (pdf) is expressed as a convex linear combination of one or more Gaussian densities ([13], Chapter 5). GMMs are capable of approximating any continuous density with arbitrary precision, provided that the model contains a sufficiently large number of mixture components [14,15,16,17,18].

For a given financial asset, the distribution of log-returns in (1) can be approximated by a finite mixture of Gaussian distributions, expressed as

(2) $$f\left(y;\theta \right)=\sum_{k=1}^{G}\pi_{k}\phi \left(y;\mu_{k},\sigma_{k}\right),$$

where $\pi_{k}$ denotes the mixing probabilities of the *G* components, satisfying $\pi_{k}>0$ and $\sum_{k=1}^{G}\pi_{k}=1$, and $\phi (\cdot \;;\mu_{k},\sigma_{k})$ is the Gaussian density with mean $\mu_{k}$ and standard deviation $\sigma_{k}$ for each component k=1,…,G. These can be collected in the vector $\theta ={\left[\pi ,\mu ,\sigma \right]}^{\;⊤}={\left[\pi_{1},\ldots ,\pi_{G-1},\mu_{1},\ldots ,\mu_{G},\sigma_{1},\ldots ,\sigma_{G}\right]}^{\;⊤}$ of unknown parameters that control the model’s behavior and must be estimated using either likelihood-based methods or Bayesian approaches.

The underlying assumption in modeling log-returns with the model in (2) is the absence of autocorrelation. However, this seems reasonable, as it is empirically known that log-returns over daily or longer periods typically exhibit little to no significant autocorrelation [19].

### 2.2. Estimation

Given a random sample of *n* observations ${\left\{y_{i}\right\}}_{i=1}^{n}$, the log-likelihood of a GMM with *G* components is given by

(3) $$\ell \left(\theta \right)=\sum_{i=1}^{n}\log\left\{\sum_{k=1}^{G}\pi_{k}\phi \left(y_{i};\mu_{k},\sigma_{k}\right)\right\}.$$

Maximizing the log-likelihood function (3) directly is often complicated; so, the maximum likelihood estimation (MLE) of θ is usually performed using the EM algorithm [20]. The EM algorithm, which includes a latent variable representing a component membership, is iterative and consists of two steps: the E-step (Expectation step) and the M-step (Maximization step). In the E-step, the algorithm calculates the expected membership probabilities of each data point to each of the mixture components based on the current estimates of the model parameters. In the M-step, the algorithm updates the model parameters by maximizing the likelihood of the observed data when given the estimated membership probabilities. These two steps are repeated until convergence or a maximum number of iterations is reached. Details on the use of the EM algorithm in finite mixture modeling is provided by McLachlan and Peel [21], while an in-depth treatment and further extensions can be found in McLachlan and Krishnan [22].

Following the fitting of a GMM and the determination of the MLEs of parameters $\hat{\theta }$, the density of the empirical distribution of log-returns can be calculated as

(4) $$\hat{f}\left(y;\hat{\theta }\right)=\sum_{k=1}^{G}{\hat{\pi }}_{k}\phi \left(y;{\hat{\mu }}_{k},{\hat{\sigma }}_{k}\right).$$

### 2.3. Model Selection

For the univariate GMM in (2), where each component is characterized by its own standard deviation, the model’s flexibility can be controlled by varying the number of mixture components. Therefore, selecting the appropriate number of components represents a crucial step.

A common approach for model selection in GMMs is to identify the “best” model as the one maximizing the Bayesian Information Criterion [BIC; [23]], which, for a given model M, is defined as

$${\mathrm{BIC}}_{M}=2\ell_{M}\left(\hat{\theta }\right)-\nu_{M}\log\left(n\right),$$

where $\ell_{M}\left(\hat{\theta }\right)$ indicates the maximized log-likelihood of the data sample of size *n* under model M, and $\nu_{M}$ is the number of independent parameters to be estimated.

The BIC criterion can be viewed as a form of penalized likelihood, where the penalty increases with the complexity of the model itself. Keribin [24] showed that BIC is consistent for choosing the number of components in a mixture model, under the assumption that the likelihood is bounded. However, the observed data log-likelihood in Gaussian mixtures is unbounded when the variance of at least one component approaches zero [25].

### 2.4. Bayesian Regularization

Fraley and Raftery [26] proposed a Bayesian regularization method as an effective way to avoid singularities and degeneracies in maximum likelihood estimation. In this proposal, weekly informative conjugate priors over the components’ parameters are introduced. Specifically, a Gaussian prior on the mean (conditional on the variance) is used:

$$\begin{array}{cc} \mu |\sigma^{2} & ∼N(\mu_{P},\sigma^{2}/\kappa_{P})\\ \; & \propto {\left(\sigma^{2}\right)}^{-\frac{1}{2}}\exp\left\{-\frac{\kappa_{P}}{2\sigma^{2}}{\left(\mu -\mu_{P}\right)}^{2}\right\}, \end{array}$$

and an inverse gamma prior on the variance is used as follows:

$$\begin{array}{cc} \sigma^{2} & ∼\mathrm{IG}(\nu_{P}/2,\varsigma_{P}^{2}/2)\\ \; & \propto {\left(\sigma^{2}\right)}^{-\frac{\nu_{P}+2}{2}}\exp\left\{-\frac{\varsigma_{P}^{2}}{2\sigma^{2}}\right\}, \end{array}$$

where $\mu_{P}$, $\kappa_{P}$, $\nu_{P}$, and $\varsigma_{P}^{2}$ are the hyperparameters, referring to the *mean*, *shrinkage*, *degrees of freedom*, and *scale*, respectively.

The EM algorithm is retained for model fitting, and the final parameter estimates computed are maximum a posteriori (MAP) estimates. For model selection, a slightly modified version of the BIC can be employed, replacing the maximized log-likelihood with the log-likelihood evaluated at the MAP estimates or posterior mode.

### 2.5. Moments and Risk Measures for Gaussian Mixtures

For the GMM in (2), it is possible to derive the moments of the finite mixture distribution (see [18], Section 1.2.4). Specifically, the following statistics can be computed:

$$\begin{array}{cccc} \mathrm{mean} & \; & \mu & =E\left(Y\right)=\sum_{k=1}^{G}\pi_{k}\mu_{k},\\ \mathrm{variance} & \; & \sigma^{2} & =V\left(Y\right)=\sum_{k=1}^{G}\pi_{k}\left(\sigma_{k}^{2}+{\left(\mu_{k}-\mu \right)}^{2}\right)=\sum_{k=1}^{G}\pi_{k}\left(\mu_{k}^{2}+\sigma_{k}^{2}\right)-\mu^{2},\\ \mathrm{skewness} & \; & \gamma_{3} & =\frac{E({\left(Y-\mu \right)}^{3})}{V{\left(Y\right)}^{3/2}}=\frac{\sum_{k=1}^{G}\pi_{k}\left({\left(\mu_{k}-\mu \right)}^{2}+3\sigma_{k}^{2}\left(\mu_{k}-\mu \right)\right)}{\sigma^{3}},\\ \mathrm{kurtosis} & \; & \gamma_{4} & =\frac{E({\left(Y-\mu \right)}^{4})}{V{\left(Y\right)}^{2}}=\frac{\sum_{k=1}^{G}\pi_{k}\left({\left(\mu_{k}-\mu \right)}^{4}+6\sigma_{k}^{2}{\left(\mu_{k}-\mu \right)}^{2}+3\sigma_{k}^{2}\right)}{\sigma^{4}}, \end{array}$$

provided that the component moments $\mu_{k}$ and $\sigma_{k}^{2}$ exist. Then, by the plug-in principle, substituting the GMM parameters with their MLE or MAP estimates allows for the straightforward computation of the moments of a Gaussian mixture.

Risk measures are essential tools in finance for assessing the potential losses of an asset or a portfolio. Two widely used risk measures, providing different perspectives on investment, are the *Value at Risk* (VaR) and the *Expected Shortfall* (ES). For a comprehensive treatment of quantitative risk measurements in finance, see McNeil et al. [27].

Let *L* be the random variable expressing the loss over the time period *T*, then VaR(α) is the αth upper quantile of *L*, i.e.,

VaR(α)=inf{l∈R:P(L>l)≤α}.

Since L=−Y, where *Y* is the random variable describing the log-return of an investment, VaR(α) can be defined equivalently as minus the αth quantile of *Y*, i.e.,

VaR(α)=−sup{y∈R:P(Y<y)≤α}.

If we model the distribution of log-returns using Y∼GMM(π,μ,σ) with the density given in (2), then the cumulative density function (cdf) of *Y* is given by

$$F_{Y}\left(y;\pi ,\mu ,\sigma \right)=\sum_{k=1}^{G}\pi_{k}\Phi \left(y;\mu_{k},\sigma_{k}\right),$$

where $\Phi (\cdot \;;\mu_{k},\sigma_{k})$ is the cdf of the Gaussian distribution with mean $\mu_{k}$ and standard deviation $\sigma_{k}$. Since VaR(α) corresponds to a subtraction of the α-quantile of *Y*, e.g., $q_{\alpha }$, for a GMM, it can be found numerically by solving the equation $F_{Y}\left(q_{\alpha };\pi ,\mu ,\sigma \right)-\alpha =0$.

VaR is a popular risk measure due to its simplicity and ease of interpretation, providing the maximum potential loss over a specified time horizon with a probability equal to the confidence level 1−α. However, VaR(α) has several limitations [28,29], such as not capturing extreme losses since it does not give any information about the severity of losses which occur with a probability less than α.

To address some of VaR’s shortcomings, the Expected Shortfall (ES) is proposed to represent the expected loss given that the loss exceeds VaR, i.e.,

$$\mathrm{ES}\left(\alpha \right)=E\left[L|L\geq \mathrm{VaR}\left(\alpha \right)\right]=\frac{1}{\alpha }\int_{0}^{\alpha }\mathrm{VaR}\left(u\right)ụ.$$

Thus, the ES provides a more comprehensive view of investment risk, making it particularly valuable during periods of high market volatility or in portfolios with significant exposure to extreme risks. ES(α) can be obtained by numerical integration from the solution for VaR(α). Alternatively, as reported in (Čížek et al. [30], Section 2.3.2), the ES of a GMM can be computed directly as

$$\mathrm{ES}\left(\alpha \right)=-\frac{1}{\alpha }\sum_{k=1}^{G}\pi_{k}\left(\mu_{k}\Phi \left(q_{\alpha };\mu_{k},\sigma_{k}\right)-\sigma_{k}\phi \left(q_{\alpha };\mu_{k},\sigma_{k}\right)\right),$$

where $\phi (\cdot \;;\mu_{k},\sigma_{k})$ is the pdf of the Gaussian distribution with mean $\mu_{k}$ and standard deviation $\sigma_{k}$.

Recently, Morkunaite et al. [31] proposed estimating VaR through simulations from a two-component GMM. Similarly, Seyfi et al. [32] introduced a Monte Carlo algorithm for calculating VaR and ES based on GMMs for an investment portfolio. In contrast, our approach is more flexible and does not require simulations to derive such risk measures.

### 2.6. Entropy Estimation

Estimating (differential) entropy for finite mixture distributions is a challenging problem that has received substantial attention in the recent literature. Among the most notable contributions, Huber et al. [33] provided exact lower and upper bounds for the entropy of GMMs, though they only offer a second-order Taylor expansion as an approximation for the entropy itself, which proves to be quite inaccurate. An improved approximation can be obtained using the *Unscented Transformation* (UT) approach of Goldberger and Aronowitz [34], where the required integral is approximated by computing an average of the log-density evaluated over the set of so-called *sigma-points*. More recently, Robin and Scrucca [35] proposed a general solution for entropy estimation applicable to any type of mixture.

We recall that, from a generative point of view, GMMs can be re-expressed as a hierarchical model by introducing the multinomial discrete latent variable Z∈{1,…,G} and writing

$$\begin{array}{cc} Z & ∼M(1,\pi )\\ Y|(Z=k) & ∼N(y;\mu_{k},\sigma_{k}), \end{array}$$

and then, by the law of total expectation, the entropy can be written as

$$\begin{array}{cc} H(Y) & =-E_{Z}\left[E_{Y|Z}\left[\log f(y)\right]\right]=-\sum_{k=1}^{G}\pi_{k}E_{Y}\left[\log f(y)|Z=k\right]\\ \; & =-\sum_{k=1}^{G}\pi_{k}\int_{Y}\phi \left(y;\mu_{k},\sigma_{k}\right)\;\log\phi \left(y;\mu_{k},\sigma_{k}\right)\;dy. \end{array}$$

Based on this formulation, Robin and Scrucca [35] showed a simple closed-formula mixture-based estimator of the (differential) entropy for samples of size *n* as follows:

(5) $$\hat{H}\left(Y\right)=-\frac{1}{n}\sum_{i=1}^{n}\log\left\{\sum_{k=1}^{G}{\hat{\pi }}_{k}\;\phi \left(y_{i};{\hat{\mu }}_{k},{\hat{\sigma }}_{k}\right)\right\},$$

with the mixture-based density estimate being evaluated on the same data points used for estimating the model’s parameters via the EM algorithm.

For a single-component GMM, it is straightforward to show that (5) is equivalent to the Gaussian entropy:

(6) $$\hat{H}\left(Y\right)=\frac{1}{2}\left(1+\log\left(2\pi \right)\right)+\log\left(\hat{\sigma }\right).$$

where $\hat{\sigma }$ is an estimate of the usual standard deviation. From (6), entropy is a monotonically increasing function of the standard deviation. However, for general univariate GMMs, as the component means move further apart, the overall standard deviation increases, but the entropy tends to plateau. For instance, consider the following two-component Gaussian mixture:

f(y)=0.5N(−μ,σ)+0.5N(μ,σ).

This model, also known as the *mixed-Gaussian distribution*, has zero mean and a variance equal to $\mu^{2}+\sigma^{2}$. Furthermore, it has been shown [36] that its entropy is given by

$$H\left(Y\right)=\frac{1}{2}\left(1+\log\left(2\pi \right)\right)+\log\left(\sigma \right)+\left(\alpha^{2}-I\right),$$

where $\left(\alpha^{2}-I\right)$ is a function of α=|μ|/σ, with values tabulated in (Michalowicz et al. [37]; Table 1).

**Figure 1:**
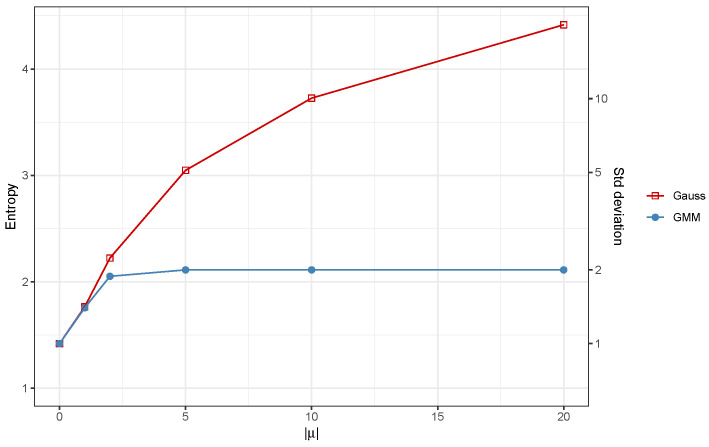
Entropy behavior for a mixed-Gaussian distribution derived from a single-Gaussian model and a two-component GMM as the component means μ diverge while the variance is held constant. Entropy values in the standard deviation scale are computed using Equation (7).

Figure 1 illustrates the behavior of the entropy for the GMM/mixed-Gaussian distribution as the component means ±μ increase, with a constant σ=1. As expected, the entropy of the GMM stabilizes once the separation between the two components reaches a certain threshold, which, in this case, occurs around |μ|=5. In contrast, if the entropy is estimated using (6) with the standard deviation of a mixed-Gaussian distribution, $\sqrt{\mu^{2}+\sigma^{2}}$, then the entropy increases monotonically as the separation between the components grows.

One advantage of using standard deviation over entropy is that standard deviation is expressed in the same unit as the log-returns, making it a more natural and easily interpretable measure. In contrast, entropy is unitless, which can complicate its interpretation in practical contexts. However, the graph in Figure 1 suggests that the entropy, even when computed from a GMM, can be converted to the standard deviation scale using the following expression:

(7) $$\sigma ={\left(2\pi \right)}^{-1/2}\exp\left(H\left(Y\right)-1/2\right).$$

This transformation allows the entropy to be expressed on the standard deviation scale and interpreted as the volatility of an equivalent Gaussian distribution with the same standard deviation.

Pele et al. [38] investigated the relationship between entropy and various market risk measures, such as Value at Risk and Expected Shortfall. However, their approach relies on an approximated entropy based on quantization, achieved by discretizing the data over a compact support. In contrast, our approach directly computes the entropy of the continuous distribution without requiring any discretization. Recently, Pichler and Schlotter [39] have provided a comprehensive overview of entropy-based risk measures, particularly focusing on entropic risk measures derived from Rényi entropy, which is a generalization of Shannon entropy.

## 3. Empirical Analyses

In this section, we present a detailed exploration of log-returns modeling using Gaussian mixture models (GMMs), with a particular focus on evaluating volatility through the entropy measure discussed in the previous section. We illustrate the practical application of GMMs in capturing the distributional characteristics of log-returns and analyze how entropy can provide insights into volatility dynamics.

Our empirical investigation covers the gold price and some major stock indices, specifically the S&P 500, FTSE, and MIB, to demonstrate the versatility and effectiveness of the proposed approach. All the data were downloaded from Yahoo Finance (https://finance.yahoo.com), accessed on 10 October 2024 using the quantmod R package [40]. For an introduction to the several tools available in R [41] for the analysis of financial data, see Tsay [42] and Hugen and Bennett [43]. The Gaussian mixture modeling discussed in this paper was performed using the mclust [44,45] and mclustAddons [46] R packages.

### 3.1. Gold Price

Gold is widely regarded as the ultimate safe-haven asset, particularly during periods of political and economic instability. While the price of gold has historically exhibited a long-term upward trend, short-term fluctuations can be significant.

We herein consider the daily gold prices (per troy ounce) in 2023 from the Commodity Exchange Inc. (COMEX), the primary futures and options market for trading metals such as gold, silver, copper, and aluminum.

**Figure 2:**
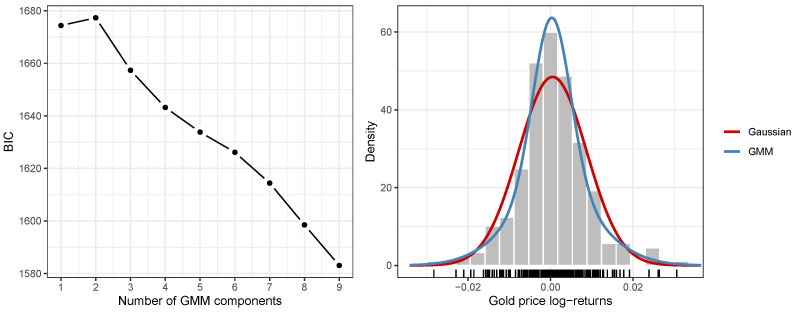
(**left**) Distribution of the 2023 daily gold price log-returns’ BIC trace for the selection of the GMM; (**right**) histogram of empirical distribution of log-returns with Gaussian density (red line) and two-component GMM density (blue line).

Figure 2 left illustrates the trace of BIC used to select the “optimal” GMM for modeling the 2023 gold price log-returns. As shown, the model with the highest BIC, indicating the best fit, is the two-component GMM. The histogram in Figure 2 right shows the empirical distribution of log-returns, with the Gaussian density (red line) and the density from the selected two-component GMM (blue line). It is evident that the single-component Gaussian model fails to adequately represent the log-returns distribution, while the two-component GMM provides a superior fit, capturing both the central region and the tails more effectively.

Table 1 compares key statistics and model metrics for both fitted models. The two-component GMM exhibits slightly lower values for both the standard deviation and entropy, with a certain degree of skewness and kurtosis compared to the single-component Gaussian model. In terms of risk measures, while the VaR at level α=0.05 is similar between the models, the ES at the same level indicates a higher expected loss for the two-component GMM. This is a consequence of the heavier tails of the implied distribution in the two-component model.

### 3.2. S&P 500

The first financial index analyzed is the S&P 500 (Standard and Poor’s 500), a stock market index that tracks the performance of 500 of the largest publicly traded companies in the US. It is calculated as the weighted average of selected companies across various sectors, with weights proportional to each company’s market capitalization. The S&P 500 is widely used as a benchmark for assessing the performance of investments.

**Figure 3:**
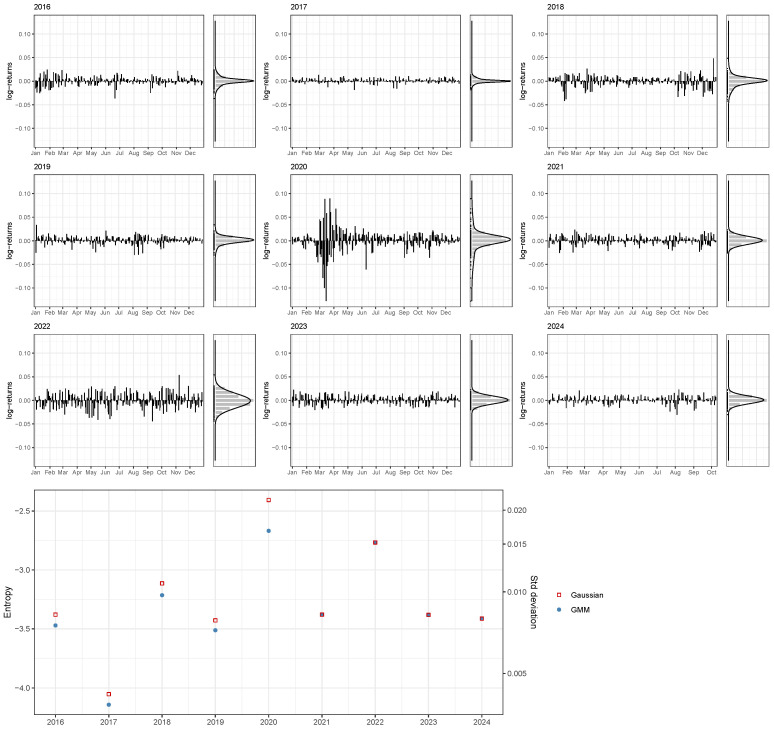
Distributions of S&P 500 daily log-returns from 2016 to early October of 2024, with histograms and corresponding GMM density estimates on the right side. The bottom graph presents yearly entropy estimates obtained using the GMM-based approach, alongside those implied by a single Gaussian component.

Figure 3 shows the daily log-returns of the S&P 500 from 2016 to early October of 2024 (at the time of writing). The vertical histograms on the right side of each year’s plot display the empirical distribution of daily log-returns, alongside the corresponding GMM density estimate. Table 2 provides detailed statistics for the estimated daily log-returns distributions of the analyzed years. From 2016 to 2020, 2-component GMMs were selected using BIC, while single Gaussian distributions were chosen for the years 2021 to 2024.

During the years 2016–2019, the log-returns remain mostly within a range of −0.05 to 0.05. There are periodic fluctuations in the market, but overall, the returns appear relatively stable. The volatility is moderate with occasional spikes, indicating some short-term market movements. The year 2020 shows significantly higher volatility, with large fluctuations in log-returns. This is consistent with the economic uncertainty caused by the COVID-19 pandemic. The volatility decreases slightly after 2020, but some fluctuations persist. The log-returns during these years are more stable than in 2020, but there are still noticeable fluctuations that reflect adjustments occurring in the stock market. During the last two years, i.e., 2023–2024, there is a clear evidence of a significantly lower volatility than in the previous years, suggesting a period of relative stability in the market.

The bottom graph in Figure 3 compares the entropy estimates derived from the GMM-based approach with those implied by a single Gaussian component. In cases where more than one component is needed to approximate the empirical distribution of log-returns, the GMM-based entropy is lower—and consequently, so is the volatility—than that suggested by the standard deviation of a single-component model. For example, in 2020, a year characterized by significant fluctuations caused by the COVID-19 pandemic, the standard deviation increased sharply, but the impact on entropy as a measure of volatility is smaller.

### 3.3. FTSE 100

The FTSE (Financial Times Stock Exchange) 100 index is a stock market index that tracks the performance of the 100 largest companies by market capitalization listed on the London Stock Exchange (LSE). The companies represent a broad range of industries, making the FTSE 100 a key indicator of the overall health of the UK stock market and economy.

**Figure 4:**
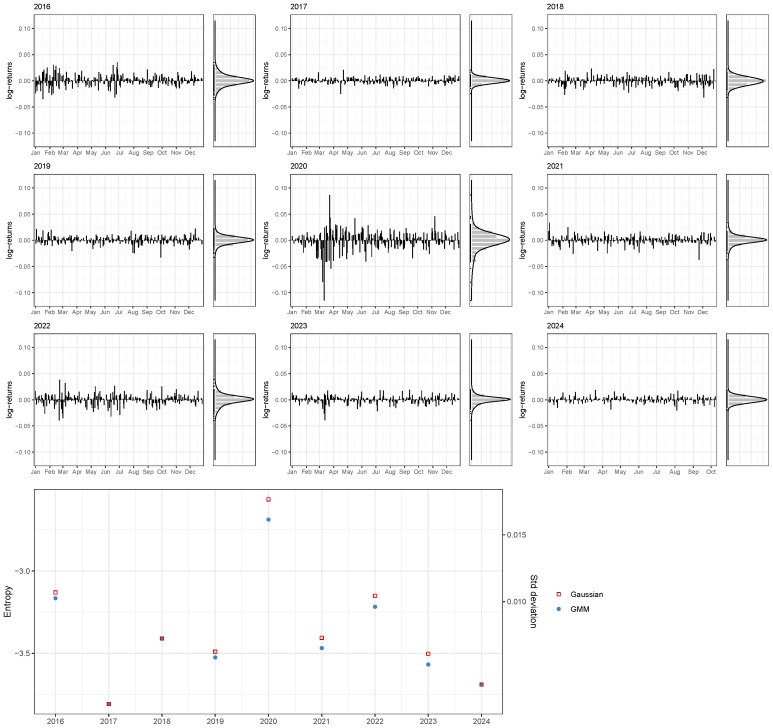
Distributions of FTSE daily log-returns from 2016 to early-October 2024, with histograms and corresponding GMM density estimates on the right side. The bottom graph presents yearly entropy estimates obtained using the GMM-based approach, alongside those implied by a single Gaussian component.

Figure 4 shows the daily distributions of log-returns of the FTSE 100 from 2016 to early October of 2024, while Table 3 provides the corresponding yearly statistics for the GMMs selected using BIC.

The pre-pandemic years, from 2016 to 2019, show a stable pattern with fluctuations within the range of −0.05 to 0.05, with most values around 0, indicating low volatility levels. In contrast, log-returns exhibit larger fluctuations during 2020, with values in the range from −0.10 to 0.10, reflecting the extreme market volatility caused by the COVID-19 pandemic. Post-pandemic years see a reduction in these fluctuations, returning to a more typical range of −0.05 to 0.05, hence aligning more closely with the pre-pandemic volatility levels. These trends are clearly visible in the bottom graph of Figure 4, where there is a clear peak in both the entropy and the standard deviation (but with an inflated value for the latter) during 2020, while both pre- and post-pandemic periods show relatively low volatility.

### 3.4. MIB

The MIB (Mercato Italiano dei Buoni del Tesoro) financial index is a benchmark index, also referred to as the FTSE MIB, that represents the performance of the top 40 Italian blue-chip companies listed on the Borsa Italiana, Italy’s main stock exchange. The index includes a wide range of sectors, financial, industrial, consumer goods and technology, providing a comprehensive picture of the Italian stock market. For this reason, investors and analysts often use the FTSE MIB to assess the health of the Italian economy and to make investment decisions.

**Figure 5:**
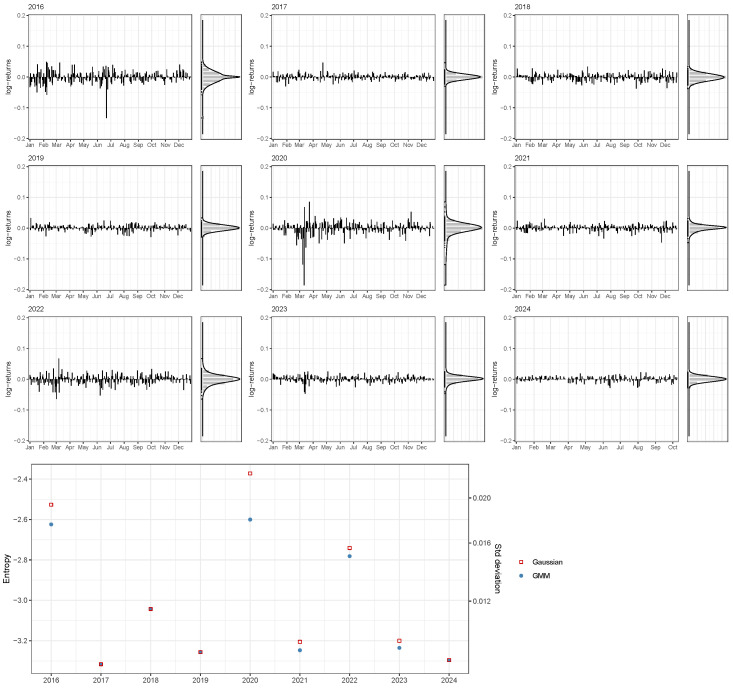
Distributions of MIB daily log-returns from 2016 to early-October 2024, with histograms and corresponding GMM density estimates on the right side. The bottom graph presents yearly entropy estimates obtained using the GMM-based approach, alongside those implied by a single Gaussian component.

Figure 5 shows the daily distribution of log-returns for the MIB from 2016 to early-October 2024, while Table 4 reports some key statistics of the stock index’s yearly distribution. Throughout these years, the log-returns fluctuate between −0.05 and 0.05, indicating relatively stable patterns. Occasionally, values exceed this range, reflecting moderate market volatility, with the exception of 2020. In that year, particularly during March and April, significant larger fluctuations occurred due to COVID-19 pandemic. During the last two years, i.e., 2023–2024, log-returns show fluctuations similar to pre-pandemic years. The bottom graph of Figure 5 illustrates the volatility patterns from 2016 to mid-2024, measured using both GMM-based entropy and Gaussian standard deviation. The years 2016, 2020, and 2022 exhibit higher volatility compared to the other years, which remain relatively stable. As noted before, entropy values are generally smaller than those obtained using Gaussian standard deviations, particularly during the years of significant market fluctuations.

## 4. Conclusions

In this paper, we present an entropy-based approach for the volatility analysis of financial log-returns using Gaussian mixture models (GMMs). This framework extends traditional volatility measures by exploiting the flexibility of GMMs to model complex, asymmetric, and heavy-tailed distributions of log-returns, and by incorporating entropy as a measure of volatility. Our proposal overcomes the limitations of standard volatility measures, such as standard deviation, which assumes normally distributed log-returns, and it is known to be sensitive to the presence of outliers. By directly estimating the entropy of a continuous distribution, the proposed approach provides a more robust and accurate assessment of financial market volatility.

Through empirical analyses on various financial assets, including gold prices and major stock indices such as the S&P 500, FTSE 100, and MIB, we demonstrate the effectiveness of our approach. The results show that the GMM-based entropy estimation captures volatility dynamics more comprehensively than traditional methods, especially during periods of high market instability, such as the 2020 COVID-19 pandemic. Additionally, the proposed framework allows for the computation of risk measures, such as Value at Risk (VaR) and Expected Shortfall (ES), using the moments derived from the GMM, which further enhances its applicability in risk management.

Looking ahead, several potential extensions emerge for future research. By adopting a moving time window within the Gaussian mixture framework, a time-varying estimate of entropy could be obtained, allowing for the modeling of the evolution of financial volatility over time. Additionally, portfolio risk measures could be derived by exploiting the linear transformation property of GMMs. Finally, following Drzazga-Szczęśniak et al. [11], future research could explore the application of entropy-based methods for predicting extreme events in financial markets, providing a potential tool for the early detection of significant market shifts.

Overall, by integrating Gaussian mixture modeling and entropy into the analysis of log-returns, we aim to provide a more accurate and robust framework for understanding financial volatility and improving the assessment of risk measures.

## Figures and Tables

**Table 1:** Statistics and model metrics for the single-Gaussian model and the two-component GMM fitted to the 2023 daily log-returns of gold prices.

Log-Likelihood	*n*	df	BIC	Mean	StDev	Skewness	Kurtosis	VaR(0.05)	ES(0.05)	Entropy
Single-component Gaussian model										
842.71	250	2	1674.4	0.0005	0.0082	0	3	0.0130	0.0165	−3.3708
Two-component Gaussian mixture model (GMM)										
852.46	250	5	1677.3	0.0005	0.0081	0.0587	4.5584	0.0129	0.0179	−3.4098

**Table 2:** Statistics for the daily distributions of S&P500 log-returns from 2016 to early October of 2024.

Year	Nobs	G	Mean	StDev	Skewness	Kurtosis	VaR	ES	Entropy	EntGauss
2016	252	2	0.0004	0.0081	−0.0979	5.0928	0.0140	0.0191	−3.4714	−3.3794
2017	251	2	0.0007	0.0041	0.4222	5.0006	0.0056	0.0082	−4.1420	−4.0527
2018	251	2	−0.0003	0.0105	−0.5603	5.3645	0.0202	0.0275	−3.2147	−3.1139
2019	252	2	0.0010	0.0076	−0.6848	5.9059	0.0121	0.0189	−3.5121	−3.4280
2020	253	2	0.0006	0.0210	−0.6926	8.2901	0.0391	0.0591	−2.6684	−2.4066
2021	252	1	0.0009	0.0082	0.0000	3.0000	0.0125	0.0159	−3.3797	−3.3798
2022	251	1	−0.0009	0.0151	0.0000	3.0000	0.0256	0.0319	−2.7669	−2.7670
2023	250	1	0.0009	0.0081	0.0000	3.0000	0.0125	0.0159	−3.3816	−3.3817
2024	195	1	0.0010	0.0079	0.0000	3.0000	0.0119	0.0152	-3.4132	−3.4134

**Table 3:** Statistics for the daily distributions of FTSE log-returns from 2016 to early-October 2024.

Year	Nobs	G	Mean	StDev	Skewness	Kurtosis	VaR	ES	Entropy	EntGauss
2016	253	2	0.0005	0.0103	0.0695	4.4766	0.0165	0.0228	−3.1660	−3.1307
2017	252	1	0.0003	0.0053	0.0000	3.0000	0.0085	0.0107	−3.8058	−3.8059
2018	253	1	−0.0005	0.0079	0.0000	3.0000	0.0135	0.0169	−3.4099	−3.4100
2019	253	2	0.0005	0.0072	−0.1689	4.5501	0.0113	0.0162	−3.5250	−3.4898
2020	253	2	−0.0006	0.0180	−0.7697	6.6364	0.0331	0.0497	−2.6886	−2.5661
2021	253	2	0.0005	0.0078	−0.1746	5.1563	0.0125	0.0183	−3.4683	−3.4062
2022	250	2	0.0000	0.0101	−0.3651	4.8830	0.0185	0.0251	−3.2176	−3.1510
2023	251	2	0.0001	0.0071	−0.3101	4.8763	0.0128	0.0174	−3.5677	−3.5028
2024	197	1	0.0003	0.0060	0.0000	3.0000	0.0095	0.0120	−3.6874	−3.6876

**Table 4:** Statistics for the daily distributions of MIB log-returns from 2016 to early-October 2024.

Year	Nobs	G	Mean	StDev	Skewness	Kurtosis	VaR	ES	Entropy	EntGauss
2016	256	3	−0.0004	0.0191	−1.2169	11.0685	0.0295	0.0446	−2.6243	−2.5268
2017	254	1	0.0005	0.0087	0.0000	3.0000	0.0138	0.0174	−3.3161	−3.3162
2018	252	1	−0.0007	0.0114	0.0000	3.0000	0.0195	0.0243	−3.0428	−3.0429
2019	251	1	0.0010	0.0092	0.0000	3.0000	0.0142	0.0181	−3.2563	−3.2563
2020	255	2	−0.0002	0.0215	−1.2875	10.2239	0.0348	0.0634	−2.6003	−2.3722
2021	256	2	0.0008	0.0096	−0.3695	4.3783	0.0167	0.0225	−3.2472	−3.2056
2022	256	2	−0.0006	0.0152	−0.2322	4.4669	0.0271	0.0369	−2.7816	−2.7410
2023	254	2	0.0010	0.0096	−0.3497	4.3518	0.0154	0.0220	−3.2354	−3.2004
2024	198	1	0.0006	0.0088	0.0000	3.0000	0.0140	0.0177	−3.2960	−3.2961

## Data Availability

Code to reproduce the analyses is available in a GitHub repository at https://github.com/luca-scr/GMMlogreturn (accessed on 22 October 2024).
